# Supplementary material for: Hot spot prediction in protein-protein interactions by an ensemble system
Source: BMC Syst Biol. 2018 Dec 31;12(Suppl 9):132. doi: 10.1186/s12918-018-0665-8 (PMC6311905; doi:10.1186/s12918-018-0665-8)
Supplement: Supplementary file 2 — All relevant MATLAB code. A simple Matlab implement of our predictor. (ZIP 19,742 kb) [file 12918_2018_665_MOESM2_ESM.zip › Additional file 2/Read ASA/ASA Property/BID/1DFJE.txt]

 NetSurfP - Protein Surface Accessibility and Secondary Structure Predictions

  

|  |  |  |
| --- | --- | --- |
|  |  | NetSurfP - Protein Surface Accessibility and Secondary Structure Predictions  Technical University of Denmark |

  


---

```
# For publication of results, please cite:
# A generic method for assignment of reliability scores applied to solvent accessibility predictions.
# Bent Petersen, Thomas Nordahl Petersen, Pernille Andersen, Morten Nielsen and Claus Lundegaard
# BMC Structural Biology 2009, 9:51 doi:10.1186/1472-6807-9-51
#
# Column 1: Class assignment - B for buried or E for Exposed - Threshold: 25% exposure, but not based on RSA
# Column 2: Amino acid
# Column 3: Sequence name
# Column 4: Amino acid number
# Column 5: Relative Surface Accessibility - RSA
# Column 6: Absolute Surface Accessibility
# Column 7: Z-fit score for RSA prediction
# Column 8: Probability for Alpha-Helix
# Column 9: Probability for Beta-strand
# Column 10: Probability for Coil
E K  1DFJE                  1    0.845 173.775   0.845   0.003   0.003   0.994
E E  1DFJE                  2    0.509  88.992  -0.840   0.058   0.017   0.925
E T  1DFJE                  3    0.426  59.031  -0.412   0.181   0.016   0.803
E A  1DFJE                  4    0.441  48.642   0.734   0.717   0.014   0.269
B A  1DFJE                  5    0.273  30.085  -1.623   0.694   0.003   0.303
E A  1DFJE                  6    0.440  48.466   1.219   0.802   0.014   0.185
E K  1DFJE                  7    0.388  79.853   1.279   0.831   0.044   0.125
B F  1DFJE                  8    0.070  14.109  -0.066   0.779   0.100   0.120
E E  1DFJE                  9    0.308  53.825   0.661   0.701   0.107   0.192
E R  1DFJE                 10    0.459 105.065   0.435   0.649   0.163   0.188
B Q  1DFJE                 11    0.234  41.721   1.419   0.478   0.309   0.214
B H  1DFJE                 12    0.182  33.106   0.435   0.159   0.516   0.325
B M  1DFJE                 13    0.132  26.393   0.359   0.022   0.359   0.619
E D  1DFJE                 14    0.298  42.956   0.296   0.020   0.205   0.775
E S  1DFJE                 15    0.297  34.762  -0.770   0.018   0.047   0.935
E S  1DFJE                 16    0.669  78.395  -0.201   0.018   0.019   0.964
E T  1DFJE                 17    0.596  82.651  -1.700   0.018   0.019   0.964
E S  1DFJE                 18    0.359  42.087  -1.235   0.016   0.005   0.979
E A  1DFJE                 19    0.535  58.957  -2.716   0.018   0.019   0.964
E A  1DFJE                 20    0.532  58.615  -1.942   0.018   0.019   0.964
E S  1DFJE                 21    0.553  64.753  -2.421   0.018   0.019   0.964
E S  1DFJE                 22    0.346  40.493  -1.829   0.053   0.005   0.942
E S  1DFJE                 23    0.479  56.080  -0.820   0.321   0.003   0.675
E N  1DFJE                 24    0.493  72.175   0.538   0.600   0.003   0.397
B Y  1DFJE                 25    0.191  40.731   0.525   0.782   0.003   0.216
B C  1DFJE                 26    0.015   2.078   1.389   0.858   0.002   0.139
B N  1DFJE                 27    0.174  25.400   0.140   0.923   0.002   0.076
E Q  1DFJE                 28    0.374  66.850   0.878   0.970   0.001   0.030
B M  1DFJE                 29    0.061  12.286   0.067   0.938   0.007   0.055
B M  1DFJE                 30    0.066  13.107   0.014   0.938   0.007   0.055
E K  1DFJE                 31    0.431  88.616   0.679   0.911   0.033   0.057
E S  1DFJE                 32    0.364  42.649   0.674   0.751   0.050   0.199
B R  1DFJE                 33    0.235  53.746   0.623   0.502   0.102   0.396
E N  1DFJE                 34    0.490  71.780  -0.838   0.428   0.171   0.402
E L  1DFJE                 35    0.350  64.158  -0.124   0.118   0.150   0.732
E T  1DFJE                 36    0.365  50.681  -0.331   0.056   0.142   0.802
E K  1DFJE                 37    0.619 127.267   0.495   0.018   0.047   0.935
E D  1DFJE                 38    0.573  82.569  -0.337   0.018   0.019   0.964
E R  1DFJE                 39    0.547 125.309   0.968   0.018   0.047   0.935
B C  1DFJE                 40    0.042   5.897  -0.901   0.019   0.141   0.840
E K  1DFJE                 41    0.336  69.177   0.980   0.020   0.205   0.775
E P  1DFJE                 42    0.561  79.634  -0.572   0.020   0.205   0.775
E V  1DFJE                 43    0.390  59.989   0.908   0.020   0.205   0.775
B N  1DFJE                 44    0.167  24.478   0.011   0.020   0.205   0.775
B T  1DFJE                 45    0.094  12.996   0.262   0.022   0.359   0.619
B F  1DFJE                 46    0.044   8.831  -0.112   0.023   0.655   0.322
B V  1DFJE                 47    0.065   9.990   0.125   0.021   0.756   0.223
B H  1DFJE                 48    0.127  23.156  -0.214   0.004   0.514   0.481
B E  1DFJE                 49    0.208  36.407  -0.030   0.004   0.138   0.858
E S  1DFJE                 50    0.335  39.297   0.184   0.018   0.047   0.935
B L  1DFJE                 51    0.208  38.140   0.174   0.600   0.003   0.397
E A  1DFJE                 52    0.691  76.115   0.786   0.694   0.003   0.303
E D  1DFJE                 53    0.407  58.591   1.153   0.694   0.003   0.303
B V  1DFJE                 54    0.024   3.704   0.465   0.802   0.014   0.185
E Q  1DFJE                 55    0.335  59.849   1.598   0.831   0.044   0.125
E A  1DFJE                 56    0.426  46.956   1.674   0.831   0.044   0.125
B V  1DFJE                 57    0.069  10.651   0.581   0.751   0.050   0.199
B C  1DFJE                 58    0.045   6.290  -0.491   0.561   0.047   0.393
E S  1DFJE                 59    0.630  73.789   0.295   0.115   0.016   0.868
E Q  1DFJE                 60    0.442  78.923  -0.092   0.016   0.005   0.979
E K  1DFJE                 61    0.673 138.477  -0.020   0.003   0.003   0.994
E N  1DFJE                 62    0.760 111.264  -0.483   0.005   0.015   0.979
B V  1DFJE                 63    0.196  30.187  -0.930   0.004   0.138   0.858
E A  1DFJE                 64    0.495  54.604  -0.003   0.005   0.262   0.733
B C  1DFJE                 65    0.108  15.093  -1.581   0.020   0.205   0.775
E K  1DFJE                 66    0.588 121.013   0.166   0.019   0.141   0.840
E N  1DFJE                 67    0.458  67.110  -0.881   0.018   0.047   0.935
E G  1DFJE                 68    0.601  47.314  -1.880   0.018   0.019   0.964
E Q  1DFJE                 69    0.562 100.445  -0.490   0.018   0.019   0.964
E T  1DFJE                 70    0.530  73.525   0.447   0.018   0.047   0.935
B N  1DFJE                 71    0.253  37.083  -0.443   0.019   0.141   0.840
B C  1DFJE                 72    0.074  10.460  -1.030   0.020   0.205   0.775
E Y  1DFJE                 73    0.307  65.649  -0.597   0.022   0.359   0.619
E Q  1DFJE                 74    0.437  77.995   0.021   0.021   0.279   0.699
B S  1DFJE                 75    0.172  20.100  -1.835   0.004   0.085   0.910
E Y  1DFJE                 76    0.665 142.153  -0.469   0.005   0.045   0.951
E S  1DFJE                 77    0.482  56.537  -1.101   0.004   0.197   0.799
E T  1DFJE                 78    0.429  59.433   0.808   0.005   0.336   0.660
B M  1DFJE                 79    0.070  14.067   0.139   0.004   0.616   0.381
E S  1DFJE                 80    0.442  51.802   1.289   0.002   0.816   0.182
B I  1DFJE                 81    0.103  19.110  -0.100   0.002   0.816   0.182
B T  1DFJE                 82    0.089  12.330   0.143   0.001   0.900   0.099
B D  1DFJE                 83    0.271  39.123   0.529   0.001   0.900   0.099
B C  1DFJE                 84    0.026   3.664   0.517   0.018   0.846   0.136
E R  1DFJE                 85    0.409  93.638   1.073   0.002   0.816   0.182
B E  1DFJE                 86    0.199  34.800   0.032   0.003   0.718   0.279
E T  1DFJE                 87    0.403  55.910   1.329   0.005   0.336   0.660
E G  1DFJE                 88    0.632  49.738  -0.996   0.004   0.085   0.910
E S  1DFJE                 89    0.590  69.207  -1.632   0.005   0.015   0.979
E S  1DFJE                 90    0.633  74.152  -0.958   0.004   0.085   0.910
E K  1DFJE                 91    0.573 117.825   0.386   0.004   0.138   0.858
E Y  1DFJE                 92    0.329  70.200  -0.079   0.004   0.085   0.910
E P  1DFJE                 93    0.367  52.120  -0.775   0.005   0.045   0.951
E N  1DFJE                 94    0.621  90.856   0.167   0.005   0.045   0.951
B C  1DFJE                 95    0.039   5.504  -1.575   0.004   0.138   0.858
E A  1DFJE                 96    0.481  53.050   1.153   0.004   0.420   0.576
B Y  1DFJE                 97    0.164  35.068  -0.641   0.004   0.616   0.381
E K  1DFJE                 98    0.483  99.353   0.765   0.022   0.552   0.426
E T  1DFJE                 99    0.398  55.189  -1.106   0.022   0.552   0.426
E T  1DFJE                100    0.408  56.617   0.472   0.004   0.514   0.481
E Q  1DFJE                101    0.558  99.623  -0.354   0.005   0.336   0.660
E A  1DFJE                102    0.482  53.083  -0.876   0.004   0.197   0.799
E N  1DFJE                103    0.442  64.767  -0.171   0.005   0.336   0.660
E K  1DFJE                104    0.301  61.895   0.743   0.004   0.420   0.576
B H  1DFJE                105    0.216  39.327   1.315   0.002   0.816   0.182
B I  1DFJE                106    0.025   4.607   0.972   0.001   0.959   0.040
B I  1DFJE                107    0.060  11.119   0.830   0.001   0.959   0.040
B V  1DFJE                108    0.017   2.582   1.358   0.001   0.959   0.040
B A  1DFJE                109    0.052   5.697   0.108   0.001   0.900   0.099
B C  1DFJE                110    0.023   3.159   0.665   0.004   0.616   0.381
E E  1DFJE                111    0.367  64.062   0.548   0.004   0.197   0.799
E G  1DFJE                112    0.651  51.242  -1.790   0.005   0.015   0.979
E N  1DFJE                113    0.506  74.122  -0.799   0.005   0.015   0.979
E P  1DFJE                114    0.414  58.761  -0.665   0.004   0.138   0.858
E Y  1DFJE                115    0.310  66.332  -0.107   0.004   0.420   0.576
B V  1DFJE                116    0.121  18.598   1.349   0.004   0.420   0.576
B P  1DFJE                117    0.073  10.345   0.011   0.004   0.420   0.576
B V  1DFJE                118    0.063   9.668   0.642   0.002   0.816   0.182
B H  1DFJE                119    0.145  26.448   0.754   0.002   0.816   0.182
B F  1DFJE                120    0.111  22.278   0.167   0.004   0.616   0.381
E D  1DFJE                121    0.323  46.487  -0.219   0.021   0.451   0.528
E A  1DFJE                122    0.429  47.265  -0.612   0.021   0.451   0.528
E S  1DFJE                123    0.386  45.251  -0.120   0.022   0.552   0.426
E V  1DFJE                124    0.484  74.375  -1.102   0.018   0.047   0.935

Explain the output.  Go back.
```

---
